# Major Depressive Disorder (MDD) and Antidepressant Medication Are Overrepresented in High-Dose Statin Treatment

**DOI:** 10.3389/fmed.2021.608083

**Published:** 2021-02-11

**Authors:** Michael Leutner, Caspar Matzhold, Alexander Kautzky, Michaela Kaleta, Stefan Thurner, Peter Klimek, Alexandra Kautzky-Willer

**Affiliations:** ^1^Clinical Division of Endocrinology and Metabolism, Department of Internal Medicine III, Medical University of Vienna, Vienna, Austria; ^2^Section for Science of Complex Systems, Center for Medical Statistics, Informatics, and Intelligent Systems (CeMSIIS), Medical University of Vienna, Vienna, Austria; ^3^Complexity Science Hub Vienna, Vienna, Austria; ^4^Department of Psychiatry and Psychotherapy, Medical University of Vienna, Vienna, Austria; ^5^Santa Fe Institute, Santa Fe, NM, United States; ^6^Institute for Applied Systems Analysis (IIASA), Laxenburg, Austria; ^7^Gender Institute, Gars am Kamp, Austria

**Keywords:** statins, depression, dyslipidemia, dosage, precision medicine

## Abstract

**Objective:** To examine the dose-dependent relationship of different types of statins with the occurrence of major depressive disorder (MDD) and prescription of antidepressant medication.

**Methods:** This cross-sectional study used medical claims data for the general Austrian population (*n* = 7,481,168) to identify all statin-treated patients. We analyzed all patients with MDD undergoing statin treatment and calculated the average defined daily dose for six different types of statins. In a sub-analysis conducted independently of inpatient care, we investigated all patients on antidepressant medication (statin-treated patients: *n* = 98,913; non-statin-treated patients: *n* = 789,683). Multivariate logistic regression analyses were conducted to calculate the risk of diagnosed MDD and prescription of antidepressant medication in patients treated with different types of statins and dosages compared to non-statin-treated patients.

**Results:** In this study, there was an overrepresentation of MDD in statin-treated patients when compared to non-statin-treated patients (OR: 1.22, 95% CI: 1.20–1.25). However, there was a dose dependent relationship between statins and diagnosis of MDD. Compared to controls, the ORs of MDD were lower for low-dose statin-treated patients (simvastatin>0– < =10 mg:OR: 0.59, 95% CI: 0.54–0.64; atorvastatin>0– < =10 mg:OR:0.65, 95%CI: 0.59–0.70; rosuvastatin>0– < =10 mg:OR: 0.68, 95% CI: 0.53–0.85). In higher statin dosages there was an overrepresentation of MDD (simvastatin>40– < =60 mg:OR: 2.42, 95% CI: 2.18–2.70, >60–80 mg:OR: 5.27, 95% CI: 4.21–6.60; atorvastatin>40– < =60 mg:OR: 2.71, 95% CI: 1.98–3.72, >60– < =80 mg:OR: 3.73, 95% CI: 2.22–6.28; rosuvastatin>20– < =40 mg:OR: 2.09, 95% CI: 1.31–3.34). The results were confirmed in a sex-specific analysis and in a cohort of patients taking antidepressants, prescribed independently of inpatient care.

**Conclusions:** This study shows that it is important to carefully re-investigate the relationship between statins and MDD. High-dose statin treatment was related to an overrepresentation, low-dose statin treatment to an underrepresentation of MDD.

## Introduction

Statins rank among the most-prescribed drugs worldwide and have significant lipid-lowering effects and hence they are usually prescribed to prevent and treat cardiovascular disease (CVD). Recently published guidelines of the European Society of Cardiology (ESC/EAS) recommend that high-risk patients, such as patients with type-2 diabetes with organ damage or patients with CVD, should have low-density lipoprotein cholesterol (LDL-cholesterol) levels lower than 55 mg/dl (1). The main mechanism of statins is inhibition of 3-hydroxy-3-methyl-glutaryl-coenenzyme A reductase (HMG-CoA reductase), which in turn inhibits synthesis of mevalonic acid, the main substrate for the synthesis of cholesterol. Cholesterol itself is the basic substance for the synthesis of essential hormones such as sex hormones or vitamin D. In this context, earlier studies have shown that statins can lower the concentrations of sex hormones for instance (2–5).

A connection between sex hormones and depression has also been demonstrated by earlier studies. Low levels of estrogen have been associated with depression in women (6–10) and withdrawal of hormone therapy for remitted perimenopausal depression has been linked to recurrence of depressive symptoms (7). Similar results have been shown for low testosterone levels in men (11–13). Earlier studies report associations between low levels of cholesterol and consequently statin usage and depression, as well as related symptoms such as lowered mood, aggression and suicidality (14–17). A potential mechanism for these associations is impaired serotonin signaling, as cholesterol is required for serotonin 1A receptor function (18–20) and hence downregulation of the serotonin 1A receptor has been linked to mood disorders such as depression (21).

More recently, the neuroinflammation hypothesis for depression has gained traction, also pointing to antidepressant effects of anti-inflammatory agents such as statins (22). Along these lines, some studies report a protective effect of statins on the development of depression (22–29). One of the major problems of the existing literature is that especially the relationship between high-dose statin treatment and MDD has yet to be investigated in detail. Data on the relationship between high-dose statin treatment and MDD in randomized controlled trials (RCTs) are particularly sparse and hence the existing literature does not clearly demonstrate that statins have an antidepressant effect, which is the main reason why they are not considered in antidepressant therapy (30). Given the paucity of data, the aim of the present study was to investigate the relationship between statins of different types and dosages and MDD.

## Methods

### Study Design

This cross-sectional retrospective analysis investigated medical claims data for the general Austrian population. Two groups [patients (1) with and (2) without statin treatment] were compared in order to investigate the relationship between statin treatment and MDD.

### Patient Population

In the present analysis, health data were investigated for all Austrians receiving health care services, i.e., ~97% of the population. These data include all diagnoses recorded during a hospital stay and data for all drug prescriptions exceeding a prescription charge of EUR 4.70. All patients alive during the observational period from January 2006 to December 2007 (*n* = 7,945,775) were analyzed, and age and sex were noted. Patients born in these years or aged >90 were excluded. The final study cohort consisted of 7,481,168 patients (3,507,903 males; 3,973,265 females). Medical prescriptions during the study period were analyzed using the Anatomical Therapeutic Chemical (ATC) Classification System codes.

### Characterization of Patients With MDD

We identified all patients diagnosed with MDD during hospital stays by using primary and secondary diagnoses, as defined by the International Classification of Diseases, 10th revision (ICD-10), codes. We classified patients as having MDD if they had a primary or secondary diagnosis of F32 (major depressive disorder, single episode) or F33 (major depressive disorder, recurrent). In order to strengthen our results, we performed a sub-analysis in which we investigated all patients who had been prescribed antidepressants (*n* = 888,596), irrespective of inpatient hospital stays. In another sub-analysis, we considered only patients with at least one hospital stay (*n* = 2,011,334), i.e., for whom diagnoses could in principle have been recorded.

### Characterization of Statin-Treated Patients and the Control Group

The statin-treated group consisted of patients prescribed one of the following six statins in at least four different quarters of a year (representing the common prescription procedure in Austria and patients' compliance) during the observational period: simvastatin (ATC-code:C10AA01), lovastatin (ATC-code:C10AA02), pravastatin (ATC-code:C10AA03), fluvastatin (ATC-code:C10AA04), atorvastatin (ATC-code:C10AA05), and rosuvastatin (ATC-code:C10AA07). Patients who had been prescribed two different types of statins over the observational period of 2 years were excluded (*n* = 5,361). The control group (non-statin-treated patients) consisted of patients to whom no statins were dispensed during the observation period. Antidepressant use was measured as the dispensing of at least one antidepressant (ATC code starting with N06A) or in combination with psycholeptics (N06CA), olanzapine (N05AH03), quetiapine (N05AH04), sulpiride (N05AL01), lithium (N05AN01), or benzodiazepine derivatives (N05BA) during the observation period.

Finally, the following groups were defined and compared:

1) Statin-treated patients vs. non-statin-treated patients.
2) Sub-analysis (independent of inpatient care): statin-treated patients on antidepressant medication vs. non-statin-treated patients on antidepressant medication.

### Average Daily Doses

Average daily doses for the drugs were calculated from the prescribed dosage, which was converted from defined daily dose to mg and divided by the number of days that were not spent in a hospital. To obtain the individual averages of the daily doses, we extracted the individual drug histories, including information on dates of received prescriptions and the corresponding dosage of the prescribed statin. The average was calculated by dividing the sum of all amounts of the administered drug by the sum of treatment days, minus the days a patient spent in hospital. The hospital days were subtracted on the assumption that the patients were treated with statins during the hospital stay.

In order to ensure precise characterization and interpretation of each substance, we defined groups according to the average daily dose for each statin, resulting in the following categories: >0–10 mg, >10–20 mg, >20–40 mg, >40–60 mg, >60–80 mg.

### Ethical Approval

This study has been approved by the ethics commission of the Medical University of Vienna (EK-Nr.: 1020/2020). A detailed statement on ethical approval is provided in the Supplementary Material. Written informed consent from the patients was not required to participate in this study in accordance with the national legislation and the institutional requirements.

### Statistical Analysis

We calculated odds ratios (ORs) between each case of statin use and diagnosis of MDD in a matched cohort analysis (each statin-treated patient was matched to three members of the control group of the same age and sex). Weighted multiple logistic regression, as described in similar analysis designs (31), was used to investigate this association while controlling for age, sex, dosage, and prescription of other medications (for diabetes and fibrates).

Patients were assigned a categorical variable for each statin comprising the respective average daily dose in mg. We controlled for use of other medications (at least four different quarters during which 20 different glucose-lowering drugs were dispensed, including metformin; three fibrates) by introducing binary dummy variables. Model quality was evaluated using the adjusted R-squared statistic, multi-collinearity via the variance inflation factor (VIF). Stratification was used to control for other diagnoses potentially associated with statin use and MDD and which could in principle act as confounding factors. These robustness tests excluded all patients with a diagnosis of ischaemic heart diseases (any code from the range I20–I25), diseases of the arteries, including arterioles and capillaries (I70–I79), stroke (I63–I64), diabetes (E10–E11), obesity (E66), hypothyroidism (E02–E03), cancer (any code from the range C00–C97), dementia in Alzheimer's disease (specific ICD code for patients with Alzheimer's disease additionally diagnosed with dementia) and Alzheimer's disease (F00, G30), pain (R52), and sleep disorder (G47). Statistical analysis was performed using standard packages of Matlab.

## Results

Of a total of 7,481,168 patients, 84,638 (1.13% of the general Austrian population) were diagnosed with MDD during a hospital stay and 888,596 (11.88% of the general Austrian population) were undergoing antidepressant therapy (prescribed independently of a hospital stay). The baseline characteristics of statin-treated patients and their age- and sex-matched control group are described in Table 1. In general, statin-treated patients (males and females) were more likely to have been diagnosed with MDD and were more commonly treated with antidepressants when compared to age- and sex-matched non-statin treated patients (OR: 1.54, CI: 1.53–1.56).

**Table 1:** Baseline characteristics of the study population, showing group size, age and the absolute and relative frequencies of depression, use of other medications (insulin, oral antidiabetics, antidepressants) and comorbid conditions for males and females in the statin-treated and in the matched control group.

	**Statin-treated patients**		**Non-statin-treated patients**	
	**Male**	**Female**	**Male**	**Female**
*N*	166,979	170,259	500,937	510,777
Age (mean +/– SD)	64.91 +/- 10.86	68.88 +/– 10.41	64.91 +/– 10.86	68.88 +/– 10.41
Depression (F32–F33)	2,947^*^ (1.76%)	6,774^*^ (3.98%)	6,907 (1.38%)	15,953 (3.12%)
Antidepressants	35,379^*^ (21.19%)	63,534^*^ (37.32%)	73,675 (14.71%)	140,846 (27.57%)
Insulin	10,697^*^ (6.41%)	11,348^*^ (6.67%)	6,281 (1.25%)	6,296 (1.23%)
Oral antidiabetics	32,639^*^ (19.55%)	30,950^*^ (18.18%)	26,605 (5.31%)	25,462 (4.98%)
CVD (I20-I25)	32,611^*^ (19.53%)	20,803^*^ (12.22%)	21,733 (4.34%)	19,631 (3.84%)
Stroke (I63, I64)	4,367^*^ (2.62%)	3,684^*^ (2.16%)	5,826 (1.16%)	5,860 (1.15%)
Diseases of arteries (I70–I79)	10,552^*^ (6.32%)	7,569^*^ (4.45%)	13,227 (2.64%)	10,415 (2.04%)
Overweight and obesity (E66)	7,164^*^ (4.29%)	7,149^*^ (4.20%)	8,384 (1.67%)	11,061 (2.17%)
Hypothyroidism (E02, E03)	1,239^*^ (0.74%)	3,277^*^ (1.92%)	2,551 (0.51%)	6,735 (1.32%)
Alzheimer's disease (F00, G30)	547^*^ (0.33%)	990^*^ (0.58%)	2,537 (0.51%)	4,381 (0.86%)
Pain (R52)	195^*^ (0.12%)	302 (0.18%)	732 (0.15%)	1,003 (0.20%)
Sleep disorders (G47)	2,806^*^ (1.68%)	951^*^ (0.56%)	4,624 (0.92%)	1,878 (0.37%)

*SD, standard deviation; CVD, cardiovascular disease, Asterisks denote statistically significant differences between statin- and non-statin treated patients;*

**p < 0.01,

* *p < 0.05*.

Supplementary Table 1 shows the baseline characteristics of the statin-treated patients with diagnosed MDD in comparison to patients with MDD without statin treatment. Depressed patients undergoing statin treatment often received antidepressant and antidiabetic therapy and displayed a higher prevalence rate of CVD, stroke, diseases of the arteries, overweight, obesity and hypothyroidism than those without statin treatment.

### Dose-Dependence of Statins on Diagnosis of MDD

**Figure 1:**
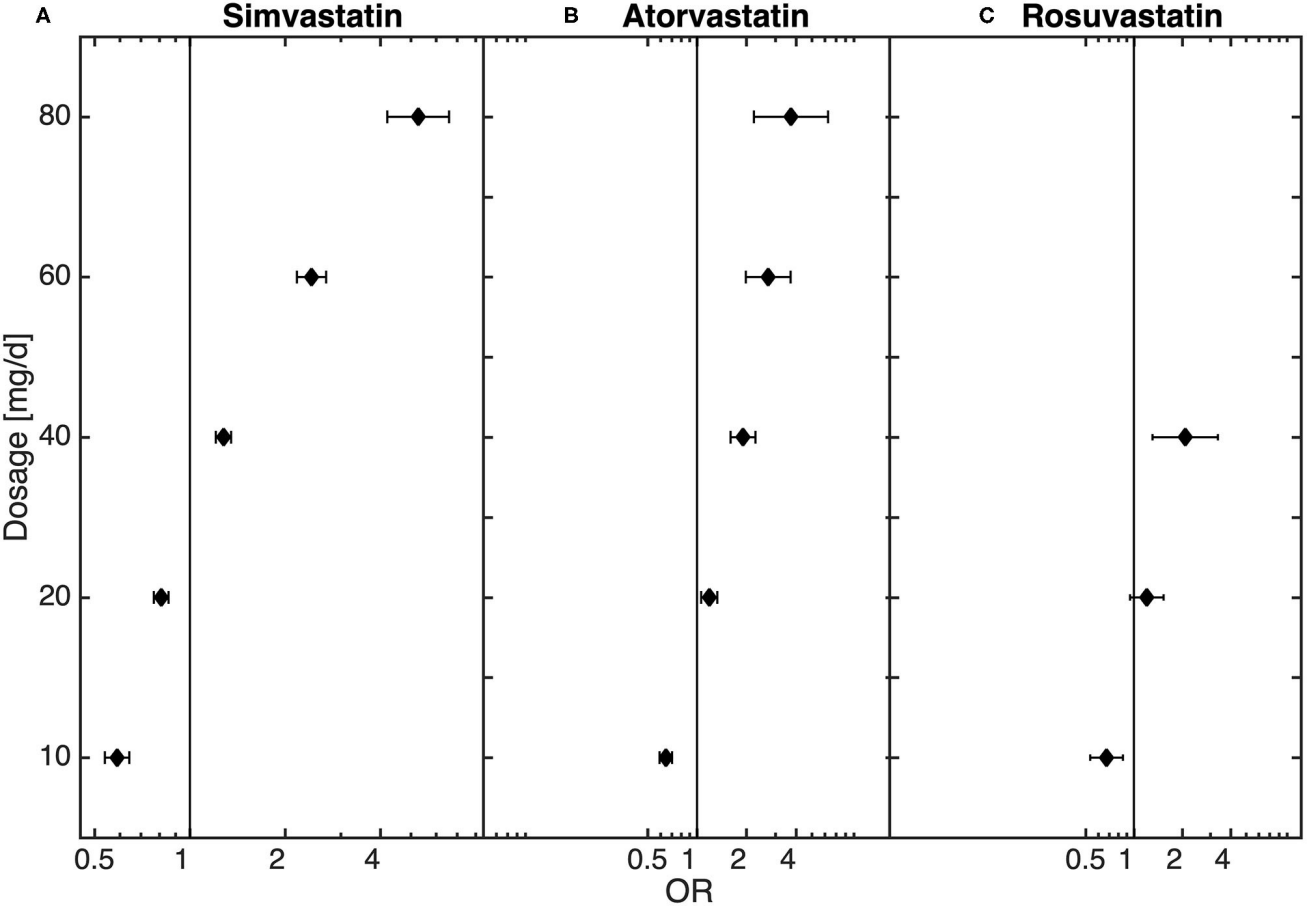
Dose-dependent relationship between **(A)** simvastatin (*n* = 214,021), **(B)** atorvastatin (*n* = 37,919), **(C)** and rosuvastatin (*n* = 8,209) and diagnosis of depression obtained from the logistic regression model.

In the general population there was an increased risk of diagnosis with depression in statin-treated patients when compared to matched controls (OR: 1.22, CI: 1.20–1.25). Further, there was a potency- and dose-dependent relationship between statins and diagnosis of MDD (see Table 2, Figure 1, Supplementary Figures 2, 3 for multiple logistic regression analyses and odds of diagnosis). In comparison to non-statin-treated patients, there was an underrepresentation of diagnosed MDD in patients receiving lovastatin in doses of >0–20 mg (0–10 mg: OR: 0.12, 95% CI: 0.05–0.27; 10–20 mg: OR: 0.62, 95% CI: 0.41–0.95). Similar results were observed for pravastatin >0–20 mg (0–10 mg: OR: 0.36, 95% CI: 0.29–0.44; 10–20 mg: OR: 0.63, CI: 0.56–0.70), simvastatin >0–20 mg (0–10 mg: OR: 0.59, 95% CI: 0.54–0.64; 10–20 mg: OR: 0.81, CI: 0.77–0.86), atorvastatin >0–10 mg (OR: 0.65, 95% CI: 0.59–0.70), rosuvastatin >0–10 mg (OR: 0.68, 95% CI: 0.53–0.85) and fluvastatin >10–60 mg. The results in Table 2 also demonstrate that the lower risk of diagnosis with MDD in low-dose statin-treated patients decreased with an increase in the potencies of statins. The OR increased with potency, the lowest OR beginning in lovastatin-treated patients. Although there was an underrepresentation of diagnosed MDD in low-dose statin treatment, the higher dosages showed an overrepresentation of MDD. Dosages of >20 mg of simvastatin (20–40 mg: OR: 1.28, 95% CI: 1.21–1.35; 40–60 mg: OR: 2.42, 95% CI: 2.18–2.70; 60–80 mg: OR: 5.27, 95% CI: 4.21–6.60), >10 mg atorvastatin (10–20 mg: OR: 1.19, 95% CI: 1.06–1.33; 20–40 mg: OR: 1.91, 95% CI: 1.60–2.27;40–60 mg: OR: 2.71, 95% CI: 1.98–3.72; 60–80 mg: OR: 3.73, 95% CI: 2.22–6.28) and rosuvastatin >20 mg (20–40 mg: OR: 2.09, 95% CI: 1.31–3.34) were related to an overrepresentation of MDD when compared to controls without statin treatment.

**Table 2:** Dose-dependent relationship between statins and diagnosis of depression.

**All**	**Lovastatin**	**Fluvastatin**	**Pravastatin**	**Simvastatin**	**Atorvastatin**	**Rosuvastatin**
>0–10 mg	0.12^**^	1.00	0.36^**^	0.59^**^	0.65^**^	0.68^**^
CI	0.05–0.27	1.00–1.00	0.29–0.44	0.54–0.64	0.59–0.70	0.53–0.85
>10–20 mg	0.62^*^	0.59^**^	0.63^**^	0.81^**^	1.19^**^	1.20
CI	0.41–0.95	0.43–0.81	0.56–0.70	0.77–0.86	1.06–1.33	0.95–1.53
>20–40 mg	1.29	0.70^**^	1.02	1.28^**^	1.91^**^	2.09^**^
CI	0.66–2.51	0.62–0.79	0.91–1.15	1.21–1.35	1.60–2.27	1.31–3.34
>40–60 mg		0.77^**^		2.42^**^	2.71^**^	
CI		0.68–0.86		2.18–2.70	1.98–3.72	
>60–80 mg		1.13		5.27^**^	3.73^**^	
CI		0.97–1.31		4.21–6.60	2.22–6.28	
Adj. R∧2	0.99	0.98	0.98	0.98	0.99	0.99
Max. VIF	1,61	1.52	1.51	1.93	1.63	1.62

** *p < 0.01;*

* *p < 0.05*.

*Bold values represent the significant ORs*.

### Robustness Test for Diseases Commonly Related to MDD

**Figure 2:**
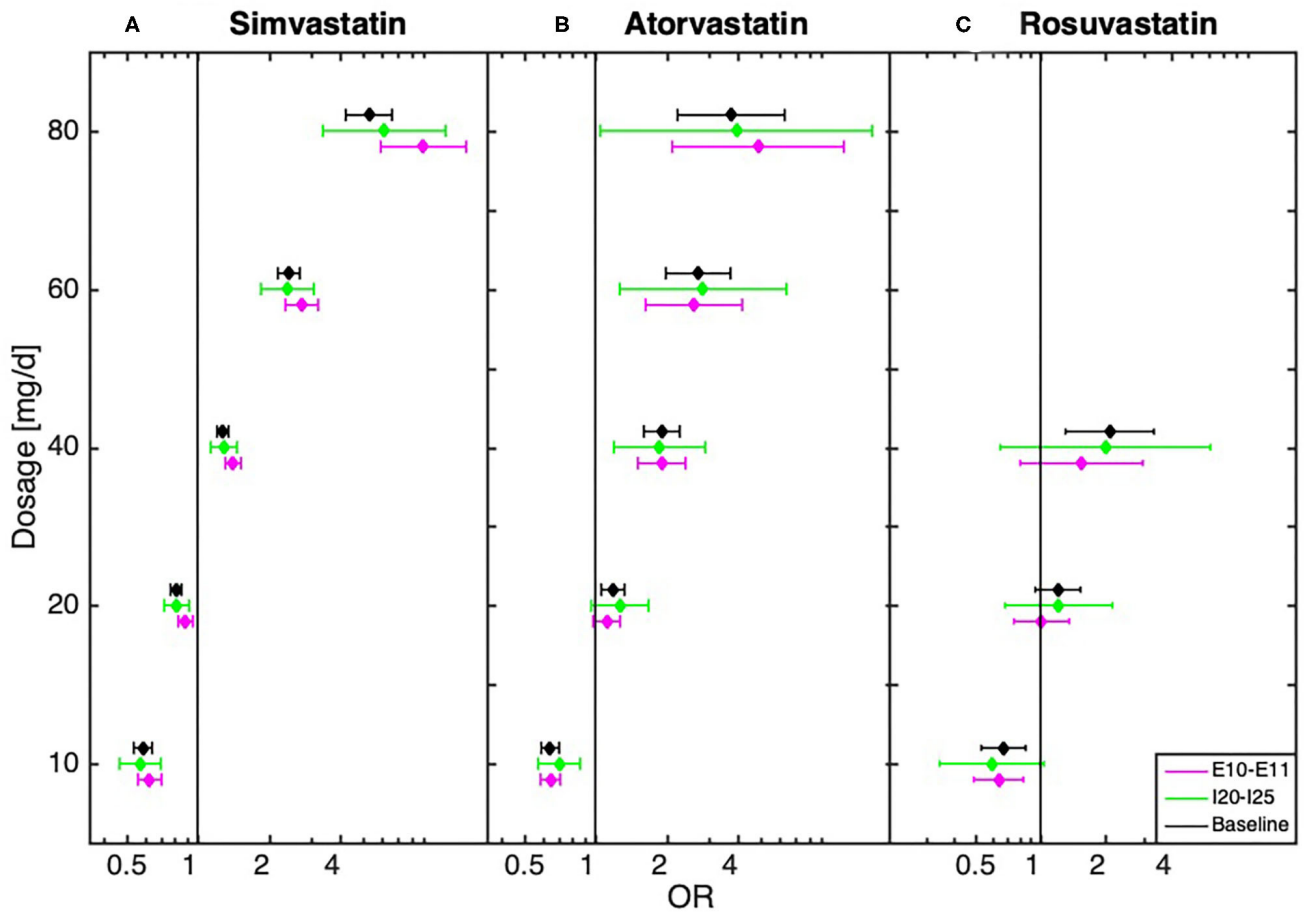
Dose-dependent relationship between **(A)** simvastatin, **(B)** atorvastatin, and **(C)** rosuvastatin and diagnosis of depression for baseline (all statin-treated patients) and after the exclusion of patients with cardiovascular disease (I20–I25, *n* = 53,414) and (*n* = 71,722)/or diabetes (E10–E11, *n* = 32,815) obtained from the logistic regression model.

We further conducted a robustness test to estimate the influence of diseases directly related to MDD, in particular ischemic heart disease, diseases of arteries, stroke, diabetes, obesity, hypothyroidism, cancer, dementia in Alzheimer's disease, Alzheimer's disease, pain, and sleep disorders. We thus tested whether the dosage-dependent MDD risk trajectories are independent of the above-mentioned disease groups (see Figure 2, Supplementary Material: baseline tests and Supplementary Figures 5–10). In these robustness tests the results showed that the observed dose dependencies followed the same trend, with an underrepresentation of diagnosed MDD in low-dose and an overrepresentation in high-dose statin-treatment. The dose-dependent relationship could also be observed in a sub-analysis that only included patients hospitalized at least once (see Supplementary Figure 1).

### Sex-Specific Analysis of the Dose-Dependent Relationship Between Statins and Diagnosis of MDD

In both male and female patients, the results shown in the general population were confirmed, demonstrating that low-dose statin treatment is related to underrepresentation of diagnosed MDD whereas high-dose statin treatment is related to overrepresentation when compared to non-statin-treated patients. Especially for high-dose atorvastatin treatment, we found that the risk of diagnosis with MDD in females was nearly double than in males. Further details can be found in Supplementary Tables 2, 3, Supplementary Figure 4.

### Sub-Analysis—Dose-Dependent Relationship Between Statin Treatment and Antidepressant Therapy

**Figure 3:**
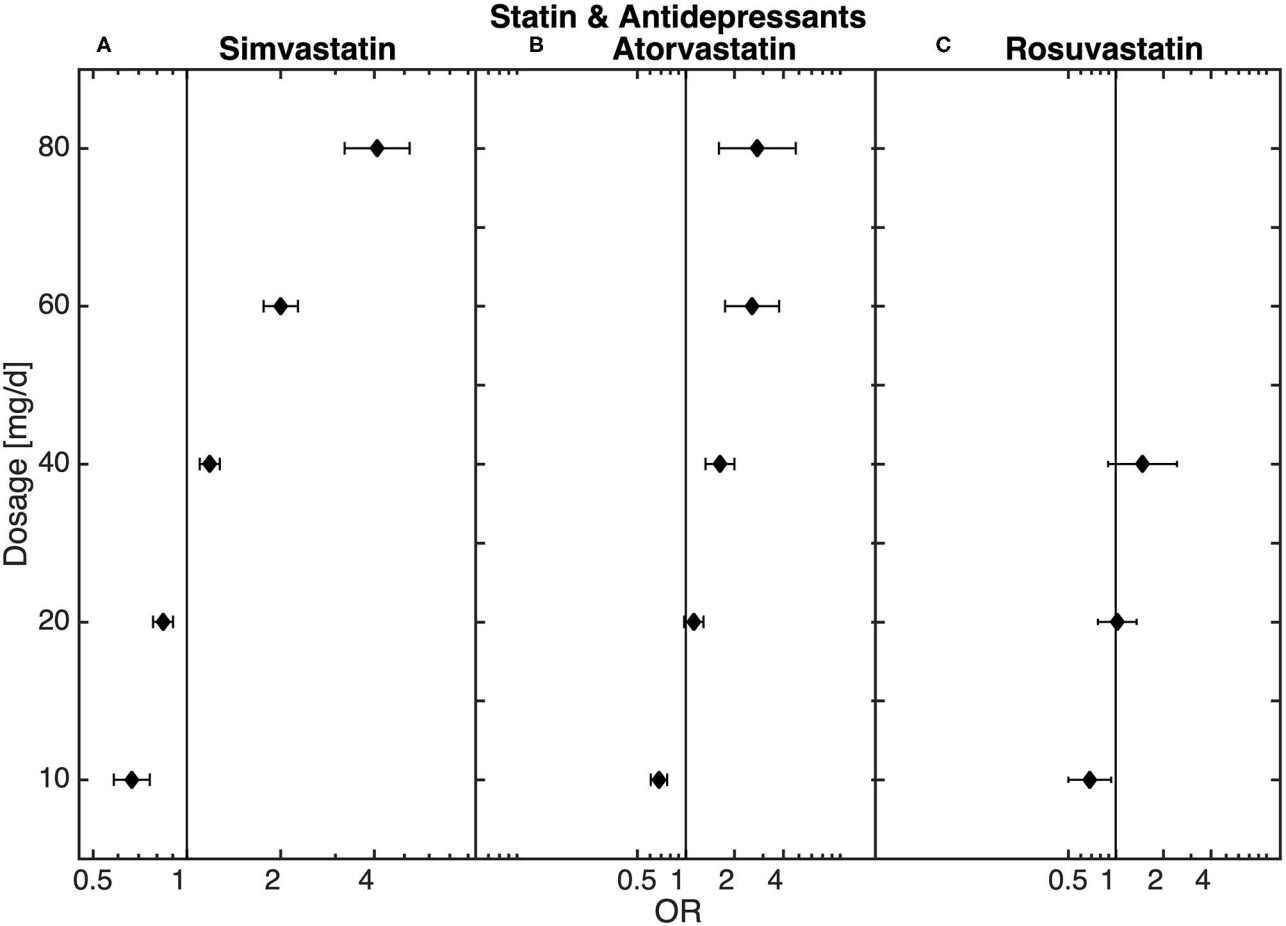
Dose-dependent relationship between **(A)** simvastatin, **(B)** atorvastatin, and **(C)** rosuvastatin and antidepressant medication (*n* = 98,913) obtained from the logistic regression model.

The final sub-analysis included 888,596 patients receiving antidepressant medication prescribed independently of hospitalization. Statin treatment was recorded for 98,913 of these patients. In the sub-analysis of all patients treated with antidepressants, similar dose-dependent results in statin-treated patients could be observed. Thus, low-dose statin treatment was related to an underrepresentation and high-dose statin treatment to an overrepresentation of antidepressant medication when compared to non-statin-treated patients (Figure 3).

## Discussion

In the present study, we investigated the relationship between different types and dosages of statins and diagnosis of MDD in comparison to non-statin-treated patients. We were able to demonstrate that there was an increased risk of diagnosed MDD in patients treated with higher doses of statins when compared to non-statin-treated patients. Interestingly, low-dose statin treatment was related to an underrepresentation of MDD when compared to non-statin-treated patients. Our findings are also supported by sex-specific results and displayed no qualitative change after exclusion of patients with diseases closely related to diagnosis of MDD, such as cardiovascular disease or diabetes, and to statin use. The dose-dependent results were also observed in a sub-analysis only including patients taking antidepressant medication prescribed independently of hospitalization.

Given the physiological mechanisms associated with statins, a relationship between statin treatment and mood disorders such as MDD seems plausible. Statins inhibit HMG-CoA reductase, which is the main mechanism for the synthesis of cholesterol, resulting in lower cholesterol levels (32) and consequently subsequent products such as sex hormones (2–5). Close relationships between low sex hormone levels and mood disorders were also suggested (7, 9, 11, 12). We have recently published evidence for a dose-dependent increased risk of diagnosis of osteoporosis in statin-treated patients and hypothesized that specifically higher dosages could inhibit the synthesis of sex hormones on a clinically relevant level and therefore advance bone resorption (33). By showing that there is also a dose-dependent relationship with diagnosed MDD, here we demonstrate for the first time that it is important to consider the different types of statins and their dosages when investigating the relationship between statin treatment and diagnosis of MDD.

Concerns about potential central nervous side-effects of statins have been raised for almost 30 years. A series of pioneering work made a case for increased depression rates as well as non-cardiovascular mortality due to violent incidents and suicide in patients with low cholesterol and thus in statin users (34–39). Interestingly, these early positive associations were rarely replicated (17), as more recent large clinical and register-based studies have generally implied no increased risk of developing MDD in statin users (22). On the contrary, protective effects have been reported and were related to neuroinflammation as a potential key mechanism of depression (40–42). Nevertheless, overall the mechanisms of putative association in either direction are insufficiently understood. Most importantly, the majority of studies have neglected different potencies and dosages of statins despite recent advances linking statins with high lipophilia and hence permeability of the blood-brain barrier but not establishing a connection between hydrophilic statins and MDD (43). Hence it is not clear whether under high-dose statin therapy a stronger downregulation of sex hormones, which are closely related to MDD, could possibly overrule the positive anti-inflammatory effect of statins on MDD.

Data regarding the dose-dependent risk of MDD in statin-treated patients are particularly sparse. For instance, in placebo-controlled clinical trials it has been shown that both low-dose lovastatin treatment (30 mg) (44) and low-dose simvastatin treatment (20 mg) (45, 46) resulted in significant relief of depressive symptoms. There are also other prospective studies demonstrating that low-dose statin treatment, such as 20 mg of atorvastatin (46, 47), could have positive effects on symptoms of depression. These results indicate that low-dose statin treatment could indeed be effective in the treatment of depression and are consequently in accordance with our results, as we found an underrepresentation of MDD in patients treated with lower doses of lovastatin (0–20 mg), pravastatin (0–20 mg), simvastatin (0–20 mg), atorvastatin (0–10 mg), and rosuvastatin (0–10 mg). There is evidence that statins can have positive effects on depression via an anti-inflammatory effect, modulation of cytokines and reduction of oxidative stress. Improved quality of life due to improved health consciousness and treatment compliance and reduced cardiovascular risk has also been linked to a potential antidepressant effect of statins (48). Nevertheless, especially the relationship to higher dosages has yet to be investigated sufficiently. That it is also important to investigate the different types of statins with their different potencies has been demonstrated by a Swedish national cohort study showing that simvastatin had a protective effect on depression, whereas atorvastatin treatment increased the risk. However, there was no detailed investigation of different dosages (25).

In the present study, higher dosages were related to an overrepresentation of MDD in statin-treated patients. These results could be confirmed by a sex-specific analysis and remain unchanged after exclusion of patients with diseases closely related to MDD. Further, similar results were observed in a sub-analysis investigating all patients taking antidepressant medication prescribed independently of hospitalization. Thus, dosage and potency may be the deciding factors for protective or risk-increasing effects. Whether a possible downregulation of hormones directly related to MDD under high-dose statin treatment could overwhelm the described positive anti-inflammatory effects of statins on MDD should be investigated in larger prospective clinical trials. The overrepresentation of diagnosed MDD in high-dose statin-treated patients is of special interest with regard to the synthesis and processing of cholesterol to essential hormones such as steroid hormones or Vitamin D. A study by Chan et al. investigated the effect of high-dose 80 mg simvastatin therapy on mood in a cohort of 140 patients with secondary progressive multiple sclerosis by conducting a 24 month, double-blind controlled trial. They showed similar results to those of our study, namely that high-dose statin treatment was related to increased severity of depressive symptoms, as measured using the Hamilton Depression Rating Scale (HAM-D) (49). Hence the fact that cholesterol is required for the serotonin 1A receptor to function (18–20) also has to be considered, since down-regulation of this receptor is closely related to mood disorders (21).

Possible interactions with the metabolization of statins should not be discounted either, as in our study females were at over double the risk of diagnosis with MDD than males when receiving high-dose atorvastatin treatment. One thus has to consider that atorvastatin is metabolized by cytochrome P450 3A4 (CYP3A4), which is also involved in the metabolism of estrogens.

Our study has both limitations and strengths. First, register-based studies are limited by the fact that there is no opportunity to precisely characterize the diagnosis of the diseases using clinical data, since we only have access to ICD codes. In the present study, the number of patients diagnosed with depression was in relation to the actual literary lower. However, a strength is that antidepressant medication was also recorded independently of hospital stays and this number reflects the actual literary and might compensate underreporting in the second group. A further limitation of the present study is that we do not have information on the duration of statin treatment and that we cannot infer causal effects using the cross-sectional study design. Additionally, it is not possible to screen the data for potential associations between different time periods of statin treatment and the relationship to major depressive disorders and/or antidepressant medication. Hence especially high-dose statin-treated patients often had a history of cardiovascular events and a higher occurrence of cardiovascular risk markers and thus MDD could therefore be a consequence of CVD (50, 51). In our dataset from 2006 to 2007 we had no access to cholesterol levels and could therefore only hypothesize that there could be a dose dependent relationship between statins and the upcoming synthesis of vital hormones (e.g., testosterone and estrogen), which are directly related to MDD. Additionally, we have no information on marital and socioeconomic status, which is also related to MDD. Also, we have no information about common side effects of statins, such as muscle symptoms or reduced exercise tolerance, and it is known that these symptoms may induce mood disorders. Further, it was not possible to provide detailed analysis of treatment adherence. In comparison to controls, statin-treated patients are characterized in the present study by a higher rate of comorbidities and it is a well-known fact that diseases such as diabetes mellitus or CHD, for instance, are closely related to the development of MDD. However, in our robustness tests, there was no qualitative change in the results after the exclusion of patients with such diagnoses.

## Conclusion

In conclusion, our results demonstrate that there exists a dose-dependent relationship between statins and diagnosis of MDD, substantiating both underrepresentation of MDD in low-dose statin treatment and increased risk of diagnosis with MDD in high-dose treatment. Considering the widespread use of statins primarily for disease prevention and increasingly stricter recommendations for tolerated cholesterol levels, these findings may be highly relevant for clinical routine across a broad spectrum of medical disciplines. This is an important and interesting approach for precision medicine in particular. Nevertheless, keeping in mind the limitations of register-based studies, prospective and longitudinal trials are urgently needed to validate our findings and further elucidate the mechanisms involved.

## Data Availability Statement

The data is not available to access because this is a consolidated research database that is only accessible for selected partners under a strict data protection policy.

## Ethics Statement

The studies involving human participants were reviewed and approved by Medical University of Vienna. Written informed consent for participation was not required for this study in accordance with the national legislation and the institutional requirements.

## Author Contributions

ML, CM, AK, MK, ST, PK, and AK-W: study design. ML, CM, and PK: data analysis. ML and CM: manuscript writing. AK-W: is the guarantor of this work. All authors read, reviewed, and approved the final manuscript.

## Conflict of Interest

The authors declare that the research was conducted in the absence of any commercial or financial relationships that could be construed as a potential conflict of interest.
