# Corrigendum: Major Depressive Disorder (MDD) and Antidepressant Medication Are Overrepresented in High-Dose Statin Treatment

**DOI:** 10.3389/fmed.2021.677866

**Published:** 2021-04-07

**Authors:** Michael Leutner, Caspar Matzhold, Alexander Kautzky, Michaela Kaleta, Stefan Thurner, Peter Klimek, Alexandra Kautzky-Willer

**Affiliations:** ^1^Clinical Division of Endocrinology and Metabolism, Department of Internal Medicine III, Medical University of Vienna, Vienna, Austria; ^2^Section for Science of Complex Systems, Center for Medical Statistics, Informatics, and Intelligent Systems (CeMSIIS), Medical University of Vienna, Vienna, Austria; ^3^Complexity Science Hub Vienna, Vienna, Austria; ^4^Department of Psychiatry and Psychotherapy, Medical University of Vienna, Vienna, Austria; ^5^Santa Fe Institute, Santa Fe, NM, United States; ^6^Institute for Applied Systems Analysis (IIASA), Laxenburg, Austria; ^7^Gender Institute, Gars am Kamp, Austria

**Keywords:** statins, depression, dyslipidemia, dosage, precision medicine

In the original article, the authors' name and surname were swapped. The correct author list appears in this article. This has been corrected in all relevant sections. The citation was corrected to “Leutner M, Matzhold C, Kautzky A, Kaleta M, Thurner S, Klimek P and Kautzky-Willer A (2021) Major Depressive Disorder (MDD) and Antidepressant Medication Are Overrepresented in High-Dose Statin Treatment. *Front. Med*. 8:608083. doi: 10.3389/fmed.2021.608083.”

The authors apologize for this error and state that this does not change the scientific conclusions of the article in any way. The original article has been updated.

