# Supplementary material for: Major Depressive Disorder (MDD) and Antidepressant Medication Are Overrepresented in High-Dose Statin Treatment
Source: Front Med (Lausanne). 2021 Feb 11;8:608083. doi: 10.3389/fmed.2021.608083 (PMC7904887; doi:10.3389/fmed.2021.608083)
Supplement: Supplementary file 1 [file Presentation_1.pdf]

Supplementary Material

Ethical approval:

We made secondary use of a research database of medical claims records that is safeguarded and maintained by the Main Association of Austrian Social Security Institutions (HVB) and in which it has been ensured that no individual is identifiable. This is a consolidated research database that is only accessible for selected partners under a strict data protection policy. Data were analyzed in full agreement and cooperation with the HVB. The data contain no names of individual patients or healthcare providers. All unique identifiers, such as social security numbers, have been removed along with patients' postal codes and dates of birth. Only district information and year of birth are available. All team members working with the data signed a confidentiality agreement and declaration of commitment ensuring that research will be undertaken in accordance with the applicable data protection regulations. Security clearance for this database signed by MUV's legal department stating that individuals are not identifiable is enclosed with this submission.

Supplementary table 1: Baseline characteristics of patients with diagnosed depression, possibly treated with or without statins

	Statin-treated patients with depression		Non-statin-treated patients with depression	
	Male	female	male	female
N	2,947	6,774	22,554	52,363
Age (mean +/- SD)	66.00 +/- 11.72	70.57 +/- 10.83	54.29 +/- 18.73	60.27 +/- 19.10
Antidepressants	2,678* (90.87%)	6,254* (92.32%)	19,104 (84.72%)	46,573 (88.95%)
Insulin	249* (8.45%)	595* (8.78%)	316 (1.40%)	727 (1.39%)
Oral antidiabetics	495* (16.80%)	1,048* (15.47%)	718 (3.18%)	1,752 (3.35%)
CVD (I20-I25)	1,336* (45.33%)	2,173* (32.08%)	2,669 (11.84%)	6,241 (11.92%)
Stroke (I63, I64)	428* (14.52%)	583* (8.61%)	1,023 (4.54%)	2,022 (3.86%)
Diseases of arteries (I70-I79)	492* (16.69%)	808* (11.93%)	1,546 (6.86%)	2,972 (5.68%)
Overweight and obesity (E66)	346* (11.74%)	926* (13.67%)	1,438 (6.38%)	4,973 (9.50%)
Hypothyroidism (E02, E03)	110* (3.73%)	542* (8.00%)	632 (2.80%)	3,527 (6.74%)
Alzheimer’s disease (F00, G30)	85 (2.88%)	218 (3.22%)	593 (2.63%)	1,810 (3.46%)
Pain (R52)	42 (1.43%)	98 (1.45%)	330 (1.46%)	896 (1.71%)
Sleep disorders (G47)	150* (5.09%)	136 (2.01%)	905 (4.01%)	1,054 (2.01%)

SD (standard deviation), CVD (cardiovascular disease), *$p<0.05$.

Supplementary figure 1: Dose-dependent relationship between simvastatin, atorvastatin and rosuvastatin and diagnosis of depression in patients with at least one hospitalization

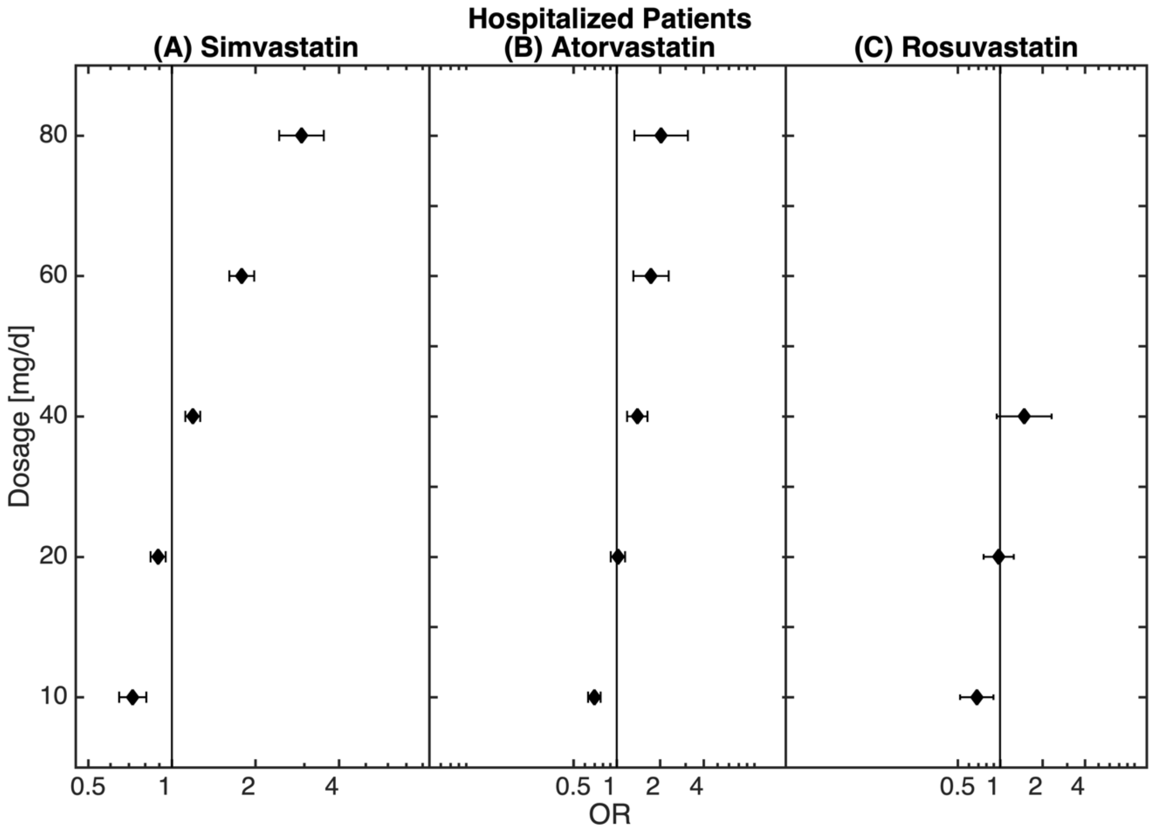

Supplementary figure 2: Dose-dependent relationship between lovastatin, pravastatin and fluvastatin and diagnosis of depression

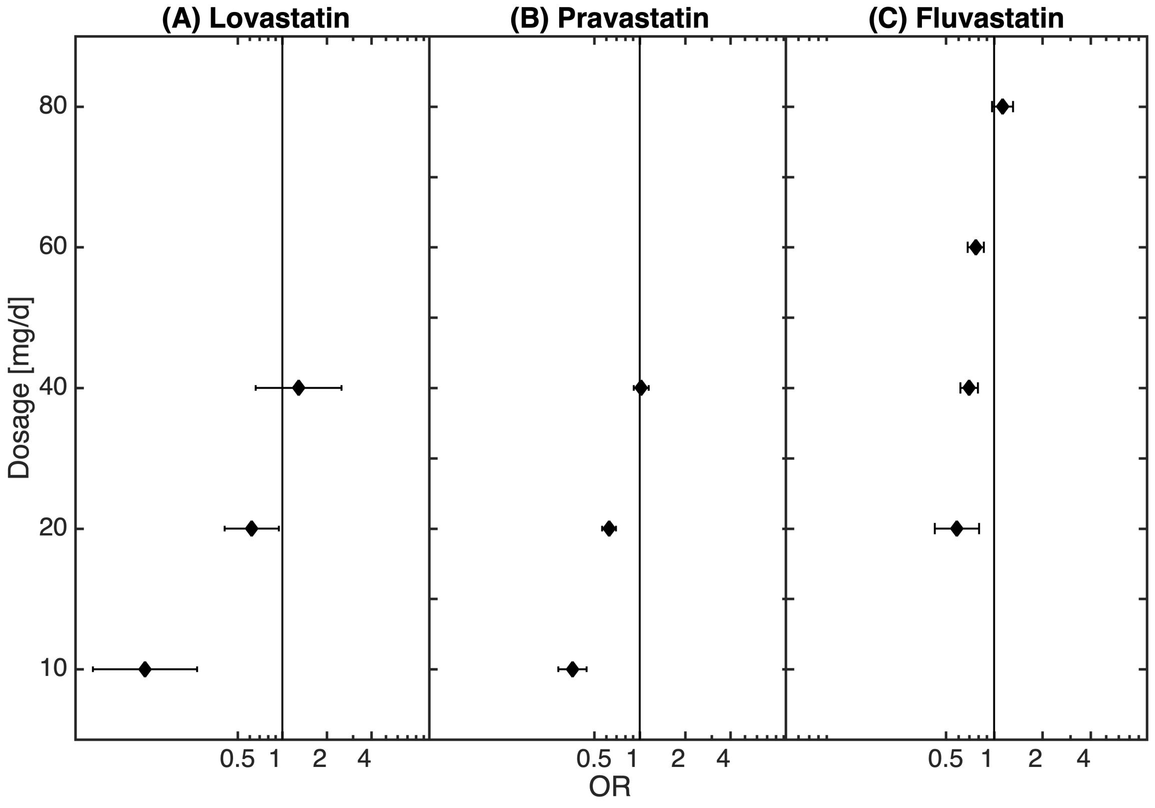

Supplementary figure 3: Dose-dependent relationship between lovastatin, pravastatin and fluvastatin and antidepressant medication

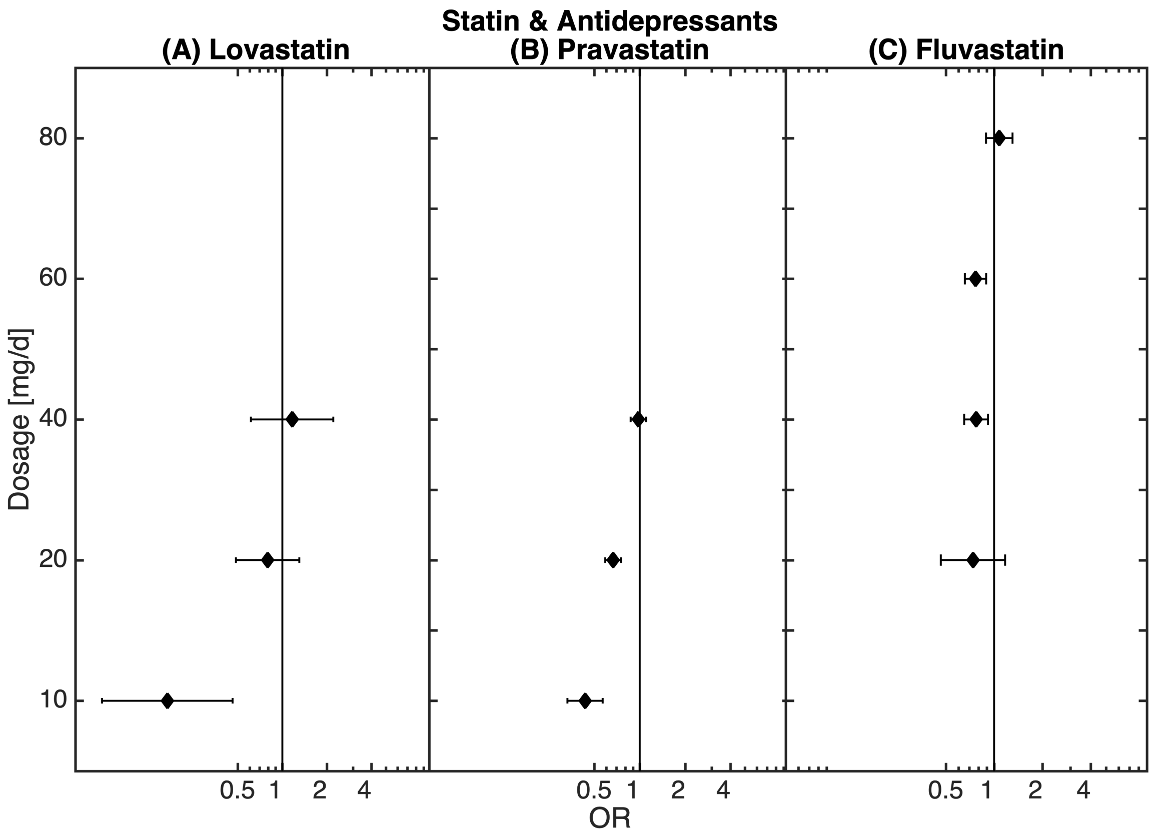

Supplementary figure 4: Sex-specific dose-dependent relationship between simvastatin, atorvastatin and rosuvastatin and diagnosis of depression (blue: male, red: female, black: all)

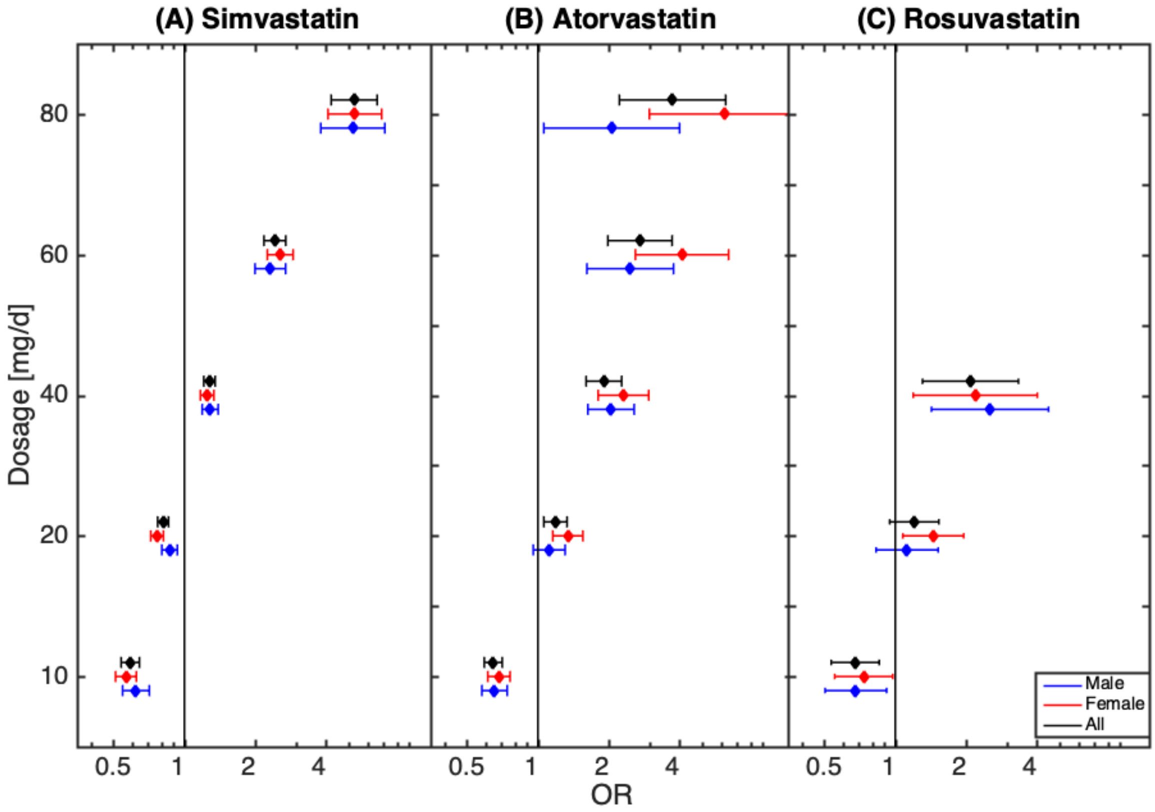

Supplementary table 2: Individual statin dosage-dependent ORs of depression (95% CI) for females and males:

Female	Lovastatin	Fluvastatin	Pravastatin	Simvastatin	Atorvastatin	Rosuvastatin
>0–10 mg	**0.14****	1.00	**0.36****	**0.56****	**0.68****	**0.73***
CI	0.06–0.33	1.00–1.00	0.28–0.47	0.51–0.62	0.61–0.76	0.55–0.98
>10–20 mg	**0.52****	**0.65***	**0.65****	**0.77****	**1.34****	1.45*
CI	0.34–0.79	0.45–0.93	0.58–0.74	0.72–0.81	1.16–1.55	1.08–1.95
>20–40 mg	1.13	**0.67****	1.05	**1.25****	**2.31****	**2.19***
CI	0.57–2.23	0.58–0.77	0.92–1.20	1.17–1.33	1.80–2.96	1.19–4.01
>40–60 mg		**0.76****		**2.55****	**4.10****	
CI		0.67–0.87		2.25–2.90	2.60–6.46	
>60–80 mg		1.08		**5.30****	**6.18****	
CI		0.91–1.29		4.07–6.89	2.98–12.83	
Adj. R^2	0.99	0.98	0.98	0.97	0.98	0.99
** p<0.01; *p<0.05						

Male	Lovastatin	Fluvastatin	Pravastatin	Simvastatin	Atorvastatin	Rosuvastatin
>0–10 mg	1.00	1.00	**0.34****	**0.62****	**0.65****	**0.68****
CI	1.00–1.00	1.00–1.00	0.25–0.46	0.55–0.71	0.58–0.74	0.50–0.92
>10–20 mg	0.76	**0.46****	**0.54****	**0.87****	**1.12****	1.12
CI	0.38–1.48	0.29–0.74	0.46–0.62	0.80–0.93	0.96–1.30	0.83–1.52
>20–40 mg	1.54	**0.78****	0.91	**1.29****	**2.04****	**2.53****
CI	0.57–4.11	0.65–0.94	0.78–1.06	1.19–1.39	1.63–2.56	1.42–4.48
>40–60 mg		**0.84***		**2.32****	**2.47****	
CI		0.70–0.99		2.00–2.69	1.61–3.77	
>60–80 mg		**1.43****		**5.20****	**2.06***	
CI		1.14–1.78		3.80–7.11	1.06–4.00	
Adj. R^2	0.98	0.97	0.97	0.97	0.97	0.98
** p<0.01; *p<0.05						

Baseline Tests:

1. Baseline (all statin-treated patients)
2. Ischaemic heart diseases (ICD code: I20–I25)
3. Stroke (ICD code: I63–I64)
4. Diseases of arteries including arterioles and capillaries (ICD code: I70–I79)
5. Diabetes mellitus type 1 and 2 (ICD code: E10–E11)
6. Overweight and obesity (ICD code: E66)
7. Hypothyroidism (ICD code: E02–E03)
8. Cancer (ICD code: C00–C97)
9. Dementia in Alzheimer's disease and Alzheimer's disease (ICD–codes: F00 and G30)
10. Pain (ICD code: R52)
11. Sleep disorders (ICD code: G47)

Robustness test:

In a multiple regression analysis we tested whether the relationship between the different kinds of statins and their dosages are independent of several diseases that are related to depression. Hence we excluded all disease groups mentioned in the baseline tests and tested the influence on OR development.

Supplementary figure 5: Dose-dependent depression risk trajectories for individual diseases in simvastatin-treated patients

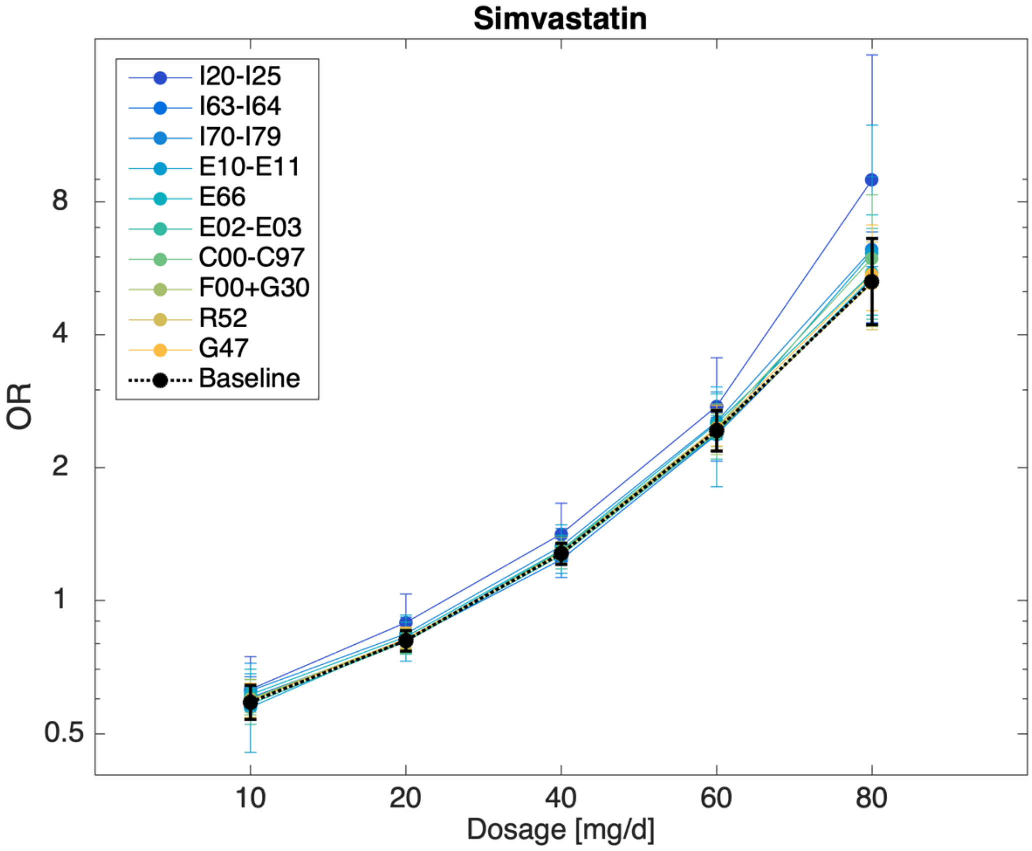

Supplementary figure 6: Dose-dependent depression risk trajectories for individual diseases in atorvastatin-treated patients

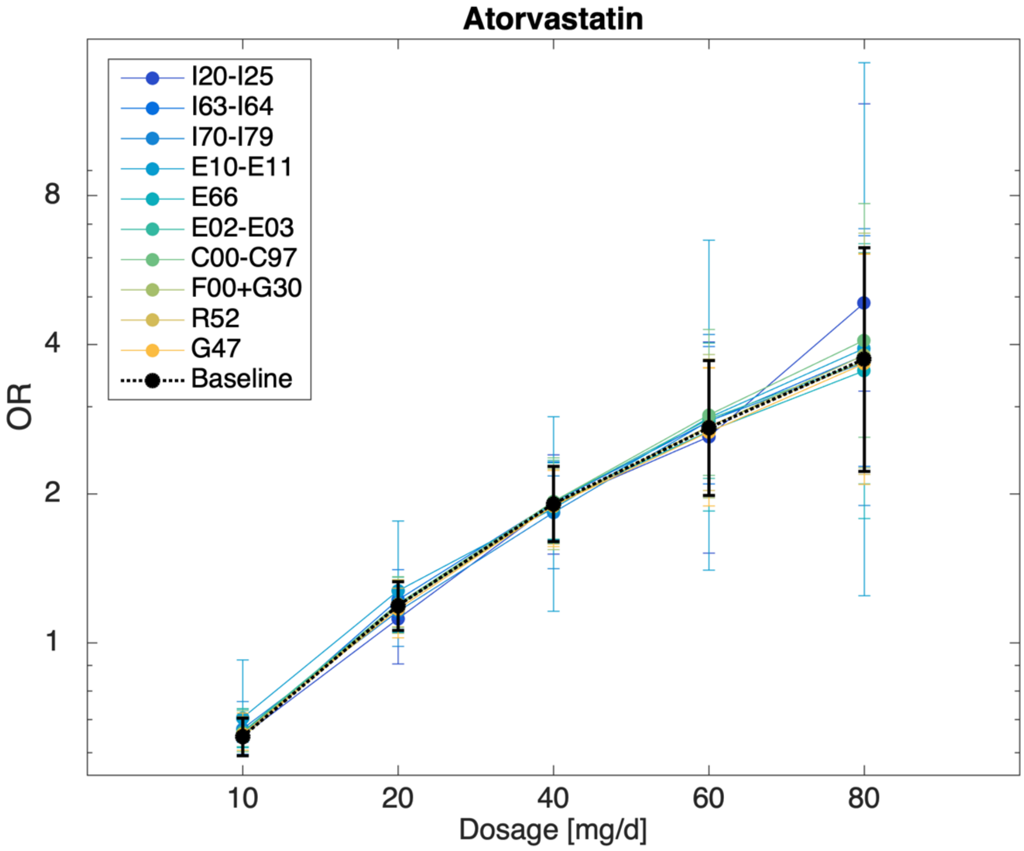

Supplementary figure 7: Dose-dependent depression risk trajectories for individual diseases in rosuvastatin-treated patients

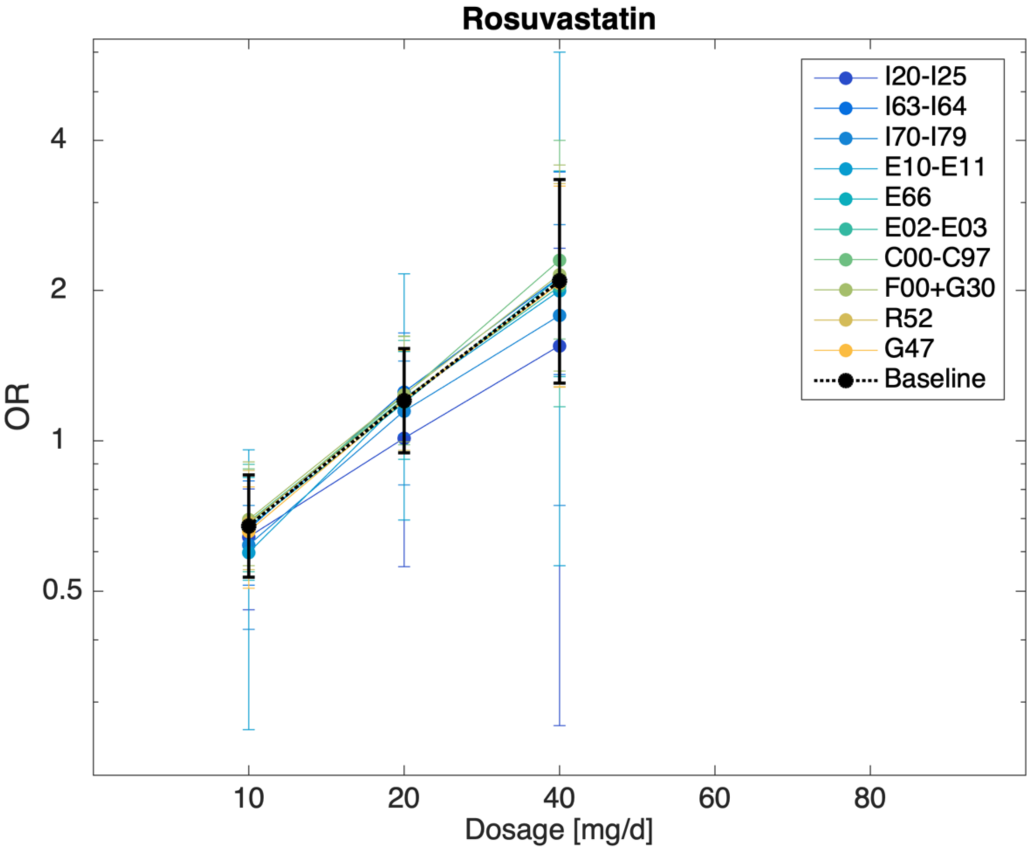

Supplementary figure 8: Dose-dependent depression risk trajectories for individual diseases in lovastatin-treated patients

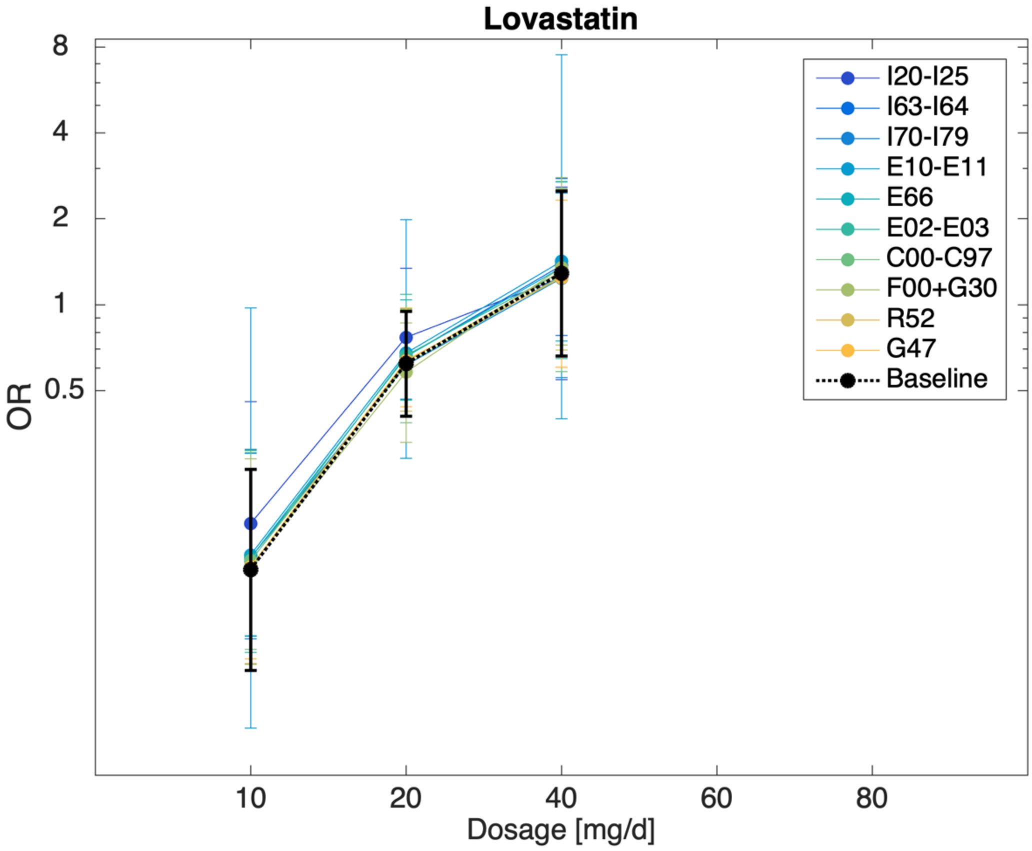

Supplementary figure 9: Dose-dependent depression risk trajectories for individual diseases in pravastatin-treated patients

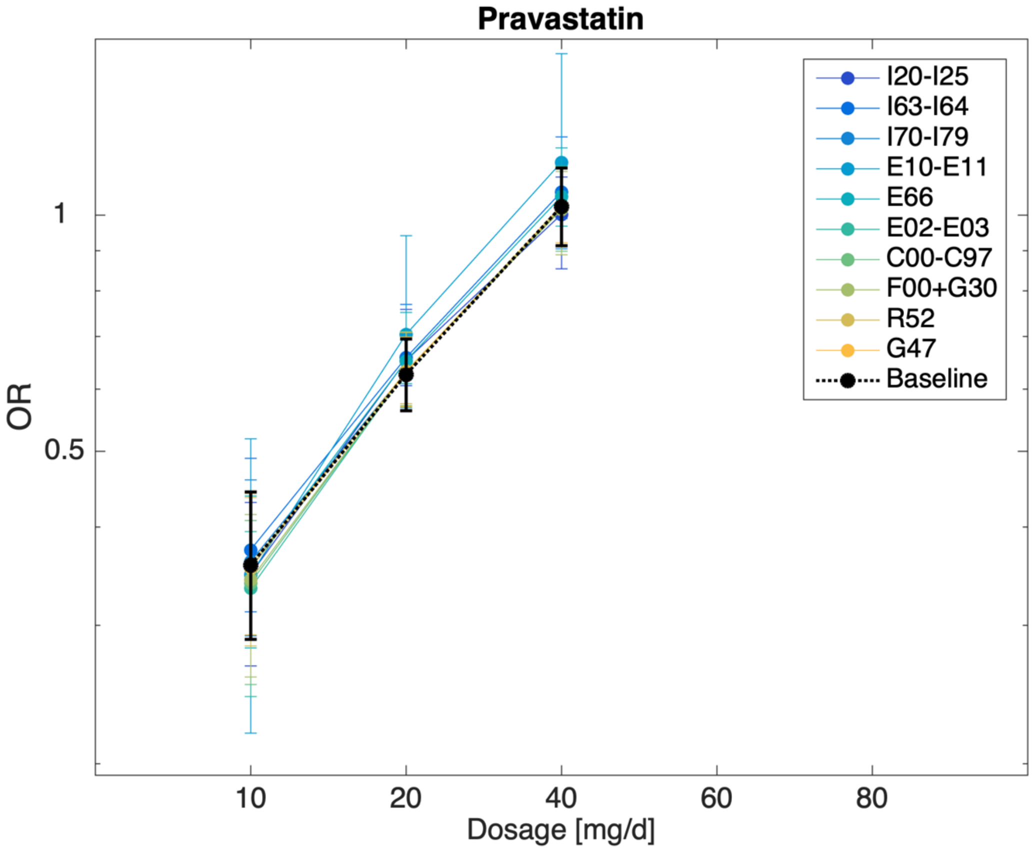

Supplementary figure 10: Dose-dependent depression risk trajectories for individual diseases in fluvastatin-treated patients

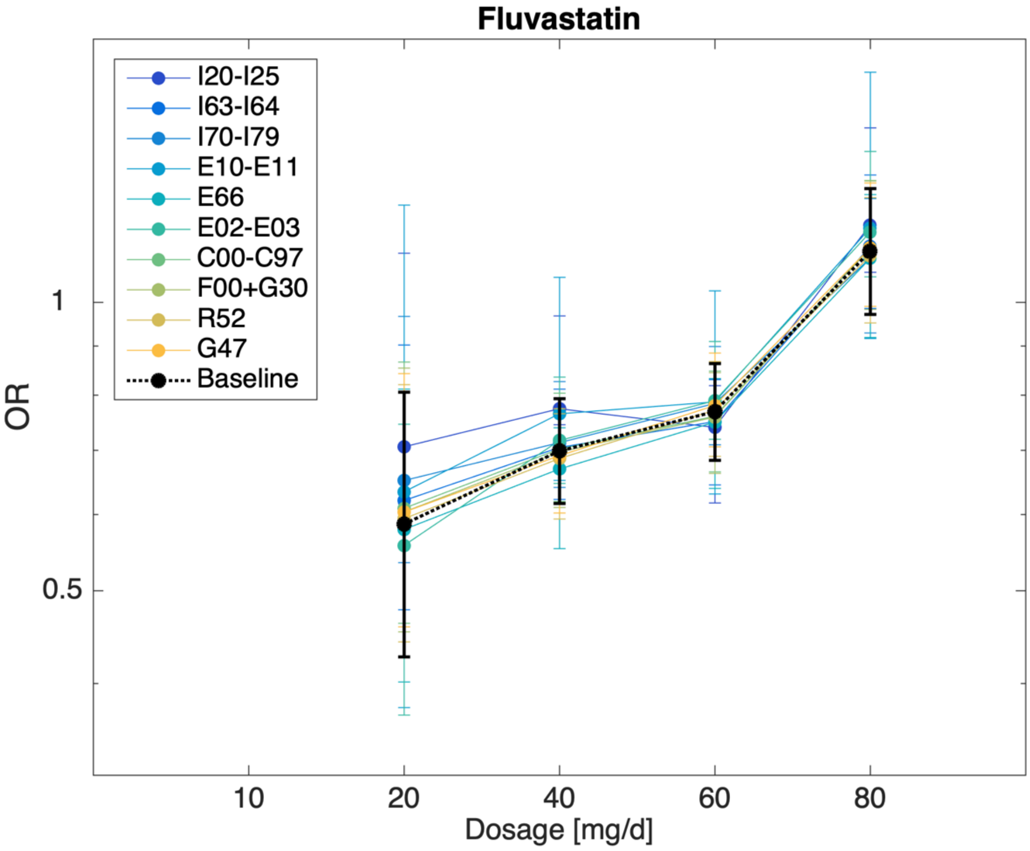

Supplementary table 3: Baseline characteristics of the study population, showing group size, age, use of other medications (insulin, oral antidiabetics, fibrates) and comorbid conditions for statin-treated patients with- or without antidepressant medication.

	Statin treated patients with antidepressant medication		Statin treated patients without antidepressant medication	
	male	female	male	female
N	35,379	63,534	131,600	106,725
Age (mean +/- SD)	65.91 +/- 11.27	69.850 +/- 10.60	64.74 +/- 10.75	68.52 +/- 10.28
Insulin	2,776* (7.85%)	4,668* (7.35%)	7,675 (5.83%)	6,543 (6.13%)
Oral-antidiabetics	6,794 (19.20%)	11.195* (17.62%)	25,845 (19.64%)	19,755 (18.51%)
Fibrates	741 (2.09%)	698 (1.10%)	2,632 (2.00%)	1,164 (1.09%)
Arthritis (M06)	89* (0.25%)	403* (0.63%)	213 (0.16%)	419 (0.39%)
CVD (I20-I25)	9,122* (25.78%)	10,021* (15.77%)	23,489 (17.85%)	10,782 (10.10%)
Stroke (I63, I64)	1.824* (5.16%)	2,207* (3.47%)	2,543 (1.93%)	1,477 (1.38%)
Diseases of arteries (I70-I79)	3,171* (8.96%)	3,594* (5.66%)	7,381 (5.61%)	3,975 (3.72%)
Obesity (E66)	1,874* (5.30%)	3,510* (5.52%)	5,290 (4.02%)	3,639 (3.41%)
Hypothyroidism (E02, E03)	421* (1.19%)	1,710* (2.69%)	818 (0.62%)	1,567 (1.47%)
Alzheimer`s disease (F00, G30)	390* (1.10%)	754* (1.19%)	157 (0.12%)	236 (0.22%)
Pain (R52)	120* (0.34%)	214* (0.34%)	75 (0.06%)	88 (0.08%)
Sleep disorders (G47)	921* (2.60%)	561* (0.88%)	1,885 (1.43%)	390 (0.37%)

SD (standard deviation), CVD (cardiovascular disease), *p<0.05.
